# The effect of pH on the acute toxicity of phenanthrene in a marine microalgae *Chlorella salina*

**DOI:** 10.1038/s41598-018-35686-9

**Published:** 2018-12-04

**Authors:** Haigang Chen, Zhe Zhang, Fei Tian, Linbao Zhang, Yitong Li, Wengui Cai, Xiaoping Jia

**Affiliations:** 1 South China Sea Fisheries Research Institute, Chinese Academy of Fishery Sciences, Guangzhou, 510300 China; 2 Guangdong Provincial Key Laboratory of Fishery Ecology and Environment, Guangzhou, 510300 China; 3 Scientific Observing and Experimental Station of South China Sea Fishery Resources and Environment, Ministry of Agriculture, Guangzhou, 510300 China

## Abstract

Phenanthrene is one of the most abundant polycyclic aromatic hydrocarbons (PAHs) found in continental shelf environment of China and is on the EPA’s Priority Pollutant list. In this study, the effects of phenanthrene on marine algal growth rate were determined after 96-h exposure at pH 6.0, 7.0, 8.0, 9.0, and 10.0 in seawater of salinity 35. Two measuring techniques to assess growth inhibition were also compared using prompt fluorescence and microscopic cell count. The results showed that the toxicity of phenanthrene increased significantly (*p* < 0.05) with decreasing pH, with the nominal concentration required to inhibit growth rate by 50%, EC_50_, decreasing from 1.893 to 0.237 mg L^−1^ as pH decreased from 9.0 to 6.0, with a decrease higher than 55% from 10.0 to 9.0. In addition, the nominal EC_50_ values calculated in this study were at the same range of some environmental concentrations of phenanthrene close to areas of crude oil exploration. Based on the two measuring techniques, the results showed that cell count and fluorescence measurement were significantly different (*p* < 0.05), and the nominal EC_50_ values calculated with cell count measurement were significantly higher than fluorescence measurement at pH 8.0, 9.0 and 10.0. In conclusion, the present studies confirmed that acidification of seawater could affect the toxicity of phenanthrene to this species of microalgae, and which encouraged further studies involving responses of marine organisms to ocean acidification.

## Introduction

Polycyclic aromatic hydrocarbon (PAHs) are a class of complex organic chemicals with two or more fused aromatic rings, which are known to be priority pollutants in the environment and can be transported to the globe from riverine runoff and long range atmospheric transport^1,2^, with the majority of inputs arising from anthropogenic sources (e.g., fossil fuel and combustion)^3^. As an important component of PAHs, phenanthrene accounted for 24% of the total PAHs^4^. Due to a relatively low molecular weight, phenanthrene would be dissolved easily in water and adsorbed to particles or lipids. For example, some seawater samples were found to contain 1.460 mg phenanthrene L^−1^ close to areas of crude oil exploration^5^. Therefore, it is more easily to be exposed to phenanthrene from both water and sediment sources for aquatic organisms^6^. Although there is not adequate evidence to assess the carcinogenicity and mutagenicity of phenanthrene, previous studies also showed phenanthrene can affect different organisms through some potential mechanism such as the aryl hydrocarbon receptor (AHR) agonists^7^, reproductive endocrine disruptor^8^ and photosynthesis stimulation^9^. But relative to the heavier PAHs such as benzo-a-pyrene^6^, information related to the toxicity of the lighter phenanthrene to marine algae was rather rare in spite of some authors surveying the toxicity of Oil and Dispersant to freshwater algae and marine algae^10,11^.

Over the past decade, ocean acidification (OA) was of profound concern for its great ecological effects on marine organisms and potential perturbations of the carbonate system^10,12^. A variety of responses of living things and their environment to OA have been studied across a global range, such as effects on organisms^13^,changes in toxicity^14^ and behavior of substances^15^. Although the natural variation has been recommended a maximum allowable deviation (0.2 pH units) for open sea^16^, OA may cause pH values more than 0.2 units of reductions and alterations in fundamental chemical balances by absorption of anthropogenic CO_2_. In addition, the range of normal sea water of 35 salinity is pH 7.8 to 8.2^17^. However, while pH value can rise up to 9.75 in the surfaces by intensive photosynthesis^18^ and to 9.67 in the shrimp pond effluent^19^. There has been rapidly growing interest in the interactions between toxicants and pH changes on marine organisms^17,18,20^. Therefore, as previous studies have shown, pH plays an important role in affecting the toxicity of chemical pollutants to organisms. This is also explained by the fact that pH may change seawater chemistry in several different ways, first is the chemical equilibrium with carbonic acid, then the chemical status of heavy metals, and the characteristics of dissolved substances^17^. For example, some authors have shown that the metal toxicity increased with increasing pH, which may resulted from the combined action by the metal ion and H^+^ at the cell surface^21,22^. Conversely, the toxicity of *p*-nitrophenol to algae decreased with increasing pH, and the least toxic effect was observed at pH 9.0^23^. The above phenomenon reflected the complex interactions between environmental factors and pollutants on the toxicity of organisms^24,25^. Phenanthrene is one of the most abundant PAHs found in continental shelf sediment of South China Sea^26^ and is on the EPA’s Priority Pollutant list (USEPA water quality criteria). Jiang *et al*.^27^ have found that phenanthrene was sufficiently high to induce acute toxicity to *Chlorella vulgaris* with EC_50_ value of 1.11 ± 0.28 mg L^−1^. Also, interactions between pH changes and the toxicity of phenanthrene can occur inevitably and deserve our great attention.

Coastal environment is considered as both sinks and secondary sources of a large input of terrigenous pollutants^28^, but aquatic organisms are exposed to various pollutants under the action of environmental factors such as temperature, salinity, pH and light in the aquatic environment^29–31^. Therefore, one of the major problems with the scientific assessment of the toxicity of environmental pollutants is to establish the impact of environmental factors during the ecotoxicity assay. And for phytoplankton, microalgae algae plays an essential role in the freshwater and marine ecosystems where it drives major ecosystem processes^32^. Any bad influence on algae is likely to affect the higher trophic organisms and may have adverse consequences for the trend of the ecological system health^32^. In this study, we performed growth inhibition tests with phenanthrene as reference chemical and with a tropical marine green algae according to OECD^33^. The objectives of this study were to find the inhibit growth rate by 50% (EC_50_) of phenanthrene on *Chlorella salina* and the effects of different pH (6.0 and 10.0) on the EC_50_) of phenanthrene. A secondary objective was to compare the difference of two measuring techniques in growth inhibition tests.

## Results

### Growth of *Chlorella salina* at different pH levels

**Figure 1:**
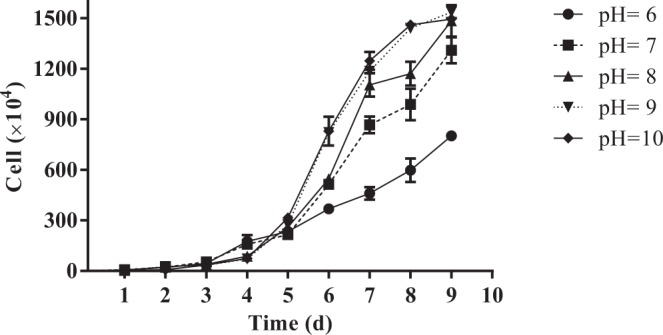
Cell growth curve as a function pH in laboratory cultures of *Chlorella salina.*

Prior to testing the effect of pH on the acute toxicity of phenanthrene, growth of *Chlorella salina* was assessed under the stated test conditions. Figure 1 showed the growth curves calculated from the average of algal cells during the time period 0 to 9 d. Exponential growth started from the 4th day of culture and still continued in the 9th day. The slowest growth rate was observed for pH 6.0, and the highest growth rates were observed at pH 9.0 and pH 10.0 closely followed by pH 8.0 and pH 7.0. Algae needed more than two times for doubling at pH 6.0 as compared to pH 9.0 and pH 10.0. At pH 10.0 and pH 9.0 the growth rate were reduced at the 9th day, therefore the cultivation of algae for the 7th day was used in the experiments. Overall, the optimum pH value was 8.0 under the culture conditions in this study.

### Effect of pH on acute phenanthrene toxicity

**Figure 2:**
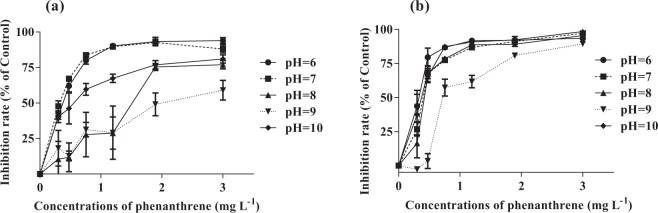
Growth rate inhibition of *Chlorella salina* after exposure to phenanthrene for 96 h at different pH levels. (**a**) cell count measurement, (**b**) fluorescence measurement.

It was observed that both cell count and chlorophyll fluorescence measurements showed the similar inhibitory effect on growth of algae increased with increasing phenanthrene concentrations at all pH levels (Fig. 2). When ANCOVA was used to investigate the combined effect with test concentration as the covariate and pH value as a fixes factor, a significant interaction for algal cells was detected (F_4,27_ = 18.51, *p* < 0.01) while both pH (F_4,27_ = 14.42, *p* < 0.01) and phenanthrene concentration (F_1,27_ = 430.85, *p* < 0.01) effects were significant (Fig. 2a). For cell counting method, pH 9.0 caused the lowest inhibition rate of 59.0% and the highest inhibition rate of 94.0% was pH 6.0 at the highest concentration of phenanthrene (3.00 mg L^−1^). Similar to cell count, a significant interaction for algal chlorophyll a was also detected (F_4,27_ = 11.90, *p* < 0.01) while both pH (F_4,27_ = 31.66, *p* < 0.01) and concentration (F_1,27_ = 1441.90, *p* < 0.01) effects were significant (Fig. 2b). For fluorimetric method, the inhibitory effect showed a clear concentration-depend response to other varying pH values. Based on the F values, phenanthrene concentration was a more dominant factor compared with pH value.

Different pH responses to phenanthrene could be observed when calculating the EC_10_ and EC_50_ values (Table 1). In five pH groups with fluorescence measurement, the EC_10_ and EC_50_ values at pH 9.0 were the significantly highest followed by pH 8.0, pH 7.0, pH 10.0 and pH 6.0, when ANOVA was performed (*p* < 0.05). Depending on the cell count measurement, the EC_50_ values also showed a significantly highest value at pH 8.0, but the significantly highest EC_10_ values was obtained at pH 9.0 (*p* < 0.05). In the paired t-test for two detection methods, the EC_10_ value showed a significant difference only at pH 7.0 between fluorescence measurement and cell count measurement (*p* < 0.05). However, a different effect occurred for EC_50_, cell count measurement resulted in a significant lower value at pH 7.0 compared to fluorescence measurement (*p* < 0.05), moreover the EC_50_ value calculated with cell count measurement was significantly higher than fluorescence measurement at pH 8.0, pH 9.0 and pH 10.0 (*p* < 0.05).

**Table 1:** EC_10_ and EC_50_ values obtained from 96 h algae acute toxicity tests of phenanthrene at different pH levels.

pH	EC_10_ (mg L^−1^)		EC_50_ (mg L^−1^)	
	Cell count	Fluorescence	Cell count	Fluorescence
6.0	0.080 ± 0.007^bc^	0.045 ± 0.013^c^	0.341 ± 0.009^c^	0.237 ± 0.039^c^
7.0	0.034 ± 0.009^c^	0.117 ± 0.012^bc^*	0.240 ± 0.222^c^	0.406 ± 0.016^b^*
8.0	0.427 ± 0.095^a^	0.168 ± 0.017^ab^	1.382 ± 0.158^b^	0.440 ± 0.030^b^*
9.0	0.197 ± 0.031^b^	0.241 ± 0.070^a^	1.893 ± 0.244^a^	0.795 ± 0.103^a^*
10.0	0.045 ± 0.009^c^	0.077 ± 0.010^bc^	0.505 ± 0.045^c^	0.355 ± 0.023^bc^*

Data were expressed as the mean ± standard error (n = 4). Symbol $\ast$ represents significant differences between cell count and fluorescence measurements, using paired t-test (*P* < 0.05). Different letters at similar at the same column indicate significant differences among the pH levels, using ANOVA (*P* < 0.05).

### Relationships between cell count and chlorophyll fluorescence

**Figure 3:**
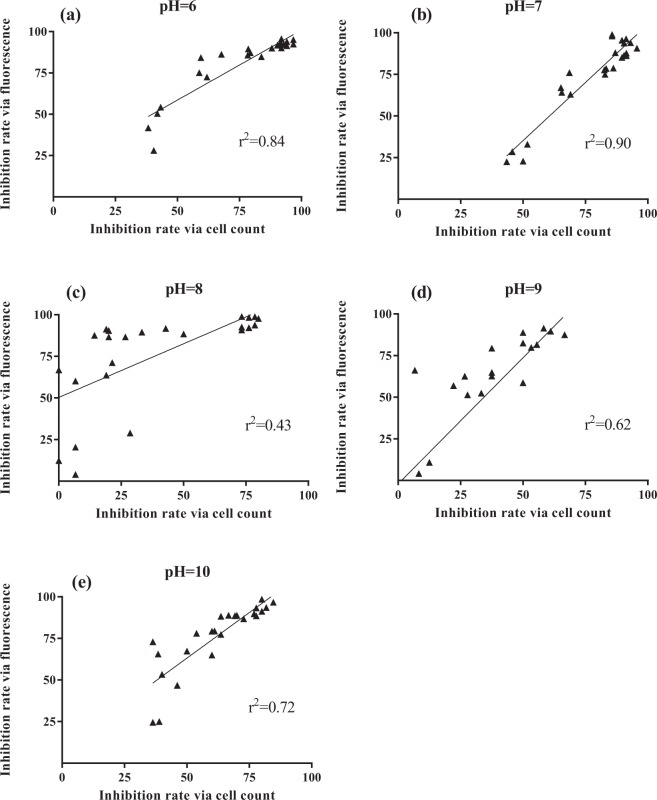
Correlation of growth inhibition rate calculated with cell count and fluorescence from phenanthrene exposure at different pH levels. (**a**) pH = 6.0, (**b**) pH = 7.0, (**c**) pH = 8.0, (**d**) pH = 9.0, (**e**) pH = 10.0.

Growth inhibition rate was determined in two ways of cell count and chlorophyll fluorescence. In results, coefficient of determination (R-square, r^2^) was 0.84 at pH 6.0, 0.90 at pH 7.0, 0.43 at pH 8.0, 0.62 at pH 9.0 and 0.72 at pH 10.0, respectively (Fig. 3). Although the relationship showed a significant positive correlation in all pH levels (*p* < 0.05), R-square values were below 0.70 at pH 8.0 and pH 9.0 (Fig. 3c,d), which showed that there were some obvious differences between cell count measurement and fluorescence measurement. However, there were high correlation to count measurement for fluorescence measurement with other treatment groups at pH 6.0, 7.0 and 10.0 (Fig. 3a,b,e).

## Discussion

Ocean acidification is a recognized problem with potentially severe impact on calcifying marine organisms such as corals and coralline algae^34^. The effects of pH on the growth of marine phytoplankton have been well documented, it suggested some species such as *Ceratium tripos*, *C*. *furca* stopped growing at a pH above 8.3 to 8.4, while others were able to grow at a pH close to 10 such as *Phaeodactylum tricornutum* and *Rhodomonas salina*, compared with documentary data on 35 species of marine phytoplankton^18^. In our study, the growth rate of *Chlorella salina* increased with increasing pH values, and the optimum pH value was 8.0 under the present culture conditions. Obviously, the results also corresponded to the normal variation of pH in the sea water of 35 salinity^17^. Similarly, Perkins proposed the limits of 6.5–9.0 and 6.7–8.5 for productive estuarine and coastal waters, respectively^35^. Apart from the above study, Schmidt and Hansen also suggested that the growth of marine phytoplankton was limited by high pH value rather than inorganic nutrients such as nitrogen and phosphorus in a standard phytoplankton growth medium (e.g. the F/2 medium)^36^. Yoo *et al*. also suggested that pH was an important main influence for the abundance of dinoflagellate when dinoflagellate abundance was correlated with environmental parameters^37^. Also, our findings in the ANCOVA for cell count and chlorophyll fluorescence also showed that there were significant differences between the growth curves under different pH level. Therefore, effects of some environmental parameters such as pH should be considered when phytoplankton growth was studied in the laboratory or natural environments.

The effects of various pollutants especially heavy metals on phytoplankton have been well documented in terms of their toxic effects and mechanisms^20,21,25,38–40^. Previous studies have indicated that the metal toxicity increased with decreasing pH for the predominance of the free metal ions in the solution with the low pH. For example, Wilde *et al*. found that the copper concentration increased from 1.0 to 19 *μ*g L^−1^ as pH decreased from 8.0 to 5.5 when achieving the inhibition of the algal growth rate by 50%^41^. This increase in pH may lead to an increase of the biousable fraction of copper, and then increase in bioavailability, finally which resulted in the increase of bioaccumulation and toxicity. Obviously the total concentration of copper inside the algae cell may then be higher than expected from the pH of the algal growth medium^42^. However, an increasing pH may decrease the bioavailability of hydrophobic contaminants, such as the apparent solubility at PH 5.5 was 3.8 times greater than at pH 7.0 in the presence of rhamnolipid^43^. Therefore, as presented in Fig. 2 and Table 1, the results of this study showed a significant decrease in phenanthrene toxicity over 96 h as the pH increased from 6.0 to 9.0. The similar phenomenon was also reported for *Chlorella vulgaris* and *Scenedesmus obliquus* following the exposure of *p*-nitrophenol at different pH levels, the toxicity of *p*-nitrophenol decreased with increasing pH and the least toxic effect was observed at pH 9.0^23^. Some reasons for the effects of pH-depended toxicity of phenanthrene and *p*-nitrophenol on algae may be suggested. The octanol-water partition coefficient (Kow) is the equilibrium ratio of concentration of a dissolved chemical between octanol and water at a specified temperature. Previous studies have indicated that pH has influence on the octanol-water partition coefficient (Kow) values^44^. Also, previous work by Borriruk wisitsak *et al*. found that the log Kow of bisphenol A changed at different pH and the bioaccumulation, sorption capacity and toxicity of bisphenol A in aquatic environments varied with changes of pH^45^. For phytoplankton, high extracellular pH may cause gross alterations in the membrane transport processes and metabolic functions involved in internal pH regulation^18,46^. Hence, variation in pH can reduce or increase the toxicity and availability of many substances, in particular weak acids and bases. Simultaneously, the EC_50_ value of phenanthrene at pH 10.0 showed a significant difference compared with pH 8.0 and pH 9.0 (Table 1 and Fig. 2a), several factors can explain the above changes, such as the complexity of pH homeostatic system^47^, high pH-induced cell cycle inhibition^48^ and the solubility and accumulation under the influence of pH^43^. In addition, another interesting finding of the study is that cell count measurements were more sensitive than fluorescence measurements only at pH 7.0 (Table 1). It is possible that cell divisions have not kept pace with chlorophyll production and the small dividing cells have weaker tolerance because of more metabolically active^49^. It is very difficult to quantify the details of these reactions currently, but in principle they play an important role with regard to both toxicity and bioaccumulation^17^.

For the algal growth inhibition test, cell density is the most simple and easy to use toxic endpoint, so cell counting with a microscope and measuring photometric absorbance are the most widely used methods^50^. The similar EC_50_ values to *Chlorella vulgaris* were obtained by Jiang *et al*.^27^ (1.11 ± 0.28 mg L^−1^ phenanthrene) and in this study (1.382 ± 0.158 mg L^−1^ phenanthrene) when using cell count measurement at the similar pH (8.0–8.2). Although cell density ultimately influences chlorophyll abundance, physiological stressors may affect chlorophyll content without affecting algae growth^51^. In this study, there were the medium degrees of correlation (r^2^ < 0.70) between cell count measurement and fluorescence measurement at pH 8.0 and pH 9.0, which showed an interesting difference. Therefore, some stressors may have affected the chlorophyll a content of microalgae without changes in cell count. The guidelines of OECD also proposed lab researchers to measure algal growth by different techniques such as particle counters, microscopic analysis, flow cytometry, fluorimetry, spectrophotometry, and colorimetry. Currently, Breuer *et al*. compared four measuring techniques for algal growth inhibition with five non-standard and two standard algal species under exposure to 3,5-dichlorophenol as reference substance, their results showed that delayed fluorescence (DF) measurement of chlorophyll was the highest sensitive detection method, followed by prompt fluorescence (PF), absorbance, and cell count^32^. As presented in Table 1, the results also showed the EC_50_ values calculated with cell count measurement were significantly higher than fluorescence measurement at pH 8.0, 9.0 and 10.0. The results in this paper are consistent with previous studies on the lower sensitivity of cell count compared with fluorescence^32^. As Chung *et al*.^52^ pointed out, phenanthrene was a phytotoxic toxicant which reduced the efficiency transfer of microalgae from light harvesting protein to PS II. Therefore, both pH and phenanthrene have effects on chlorophyll a content and may impair photosynthesis by metabolic responses of algae to some stressors^53^. With the increasing incidence of OA, the environmental factors such as pH, salinity, illumination and temperature *et al*. have become key ecological impacts of the complex interactions. In particular, the phenanthrene concentration of seawater samples (1.460 mg phenanthrene L^−1^) near areas of crude oil exploration^5^ are at the similar levels with the nominal EC_50_ values (0.237–1.893 mg L^−1^) in this study, which may exert potential risks to microalgae populations and even aquatic ecosystem.

## Conclusions

In summary, our results indicate that a reduction in pH increases the growth inhibition of *Chlorella salina* exposed to phenanthrene. Moreover, the higher sensitivity of fluorescence measurements was confirmed when comparing cell count measurement with the nominal EC_50_ values of phenanthrene. Therefore, the effect of pH-dependent and the differences between different methods need to be considered when we planned to evaluate the algal growth inhibition test under various environmental pollution conditions.

## Materials and Methods

### Preparation of chemicals and alga

Phenanthrene (purity ≥ 98%) was purchased from Sigma-Aldrich (Poole, United Kingdom). The stock solution of phenanthrene (1 g L^−1^) was prepared by dissolving an appropriate amount of phenanthrene in 30% acetone-water mixture in a brown volumetric flask and diluted with sterile sea water to target concentration before use. All other chemicals were of analytical grade and were obtained from commercial sources.

The marine microalgae *Chlorella salina* was used in the inhibition assay, which was purified and cultured with the sterilized coastal waters of the Donghai Island in the Culture Collection of Microalgae (CCM) at the south station of National Aquatic Seed Engineering of the Guangdong Hengxing Group Co., Ltd. Then the domestication and amplification of the test algae were cultured in F/2 medium^54^ prepared with the seawater (PSU = 35, pH = 8.20) from Daya Bay in Shenzhen filtered with 0.45 *μ*m Whatman filter paper, both culture medium and culture vessels were sterilized in a high temperature sterilizing oven at 121 °C for 15 min. When required, a suitable salinity was obtained by evaporation or dilution with distilled water. For stock cultures of the test organisms, which were acclimatized for 10 days at 25 °C in a light irradiated incubator (white fluorescent light 50 *μ*mol m^−2^ s^−1^) using an L:D photoperiod of 14:10 h. The cultures were hand shaken at least 4–5 times daily to ensure proper availability of nutrients to algal cells. The final incubated alga used for the test was obtained by mixing the stock culture in the logarithm stage with sterile medium and the initial cell density was 1.75 × 10^4^ cells ml^−1^.

### Toxicity tests

Algae acute toxicity tests were conducted following OECD guideline 201^33^ with glass erlenmeyer flasks with a total liquid volume of 100 mL. According to the results of pre-experiment, the pre-incubated algae was chosen to prepare exposure groups in the following of six nominal experimental concentrations, i.e. 0.300, 0.475, 0.754, 1.194, 1.893, 3.000 mg L^−1^, of phenanthrene. Algae exposed to the same volume of 0.1% acetone (carrier solvent for phenanthrene) as the above mentioned six concentrations were treated as the control. For each of exposure phenanthrene concentrations, pH was adjusted to 6.0, 7.0, 8.0, 9.0 and 10.0 with NaOH/HCl as the control. At the start of each experiment, 1.75 × 10^4^ cells ml^−1^ (N_0_) algae were prepared in sterile seawater (PSU = 35) for each exposure test with triplicate for each concentration, and air-permeable stoppers were used to seal the erlenmeyers. Then the erlenmeyers were kept for 96 h under 25 °C with a light intensity of 50 *μ*mol m^−2^ s^−1^) (14/10 h, light/dark) and were manually shaken daily. After 24, 48, 72 and 96 h of exposure, the number of cells (N_24_, N_48_, and N_72_ and N_96_) was counted with a Burker blood-counting chamber. Chlorophyll a contents were also measured fluorometrically (Turner Designs 10-AU model)^55^ after 96 h of exposure. The percentage inhibition In was determined for the each point of the test in time t from the equation:

$${\rm{In}}=({{\rm{N}}}_{c}-{{\rm{N}}}_{t})/{{\rm{N}}}_{c}\times {\rm{100}} \% \,{\rm{or}}\,{\rm{In}}=({{\rm{C}}}_{c}-{{\rm{C}}}_{t})/{{\rm{C}}}_{c}\times {\rm{100}} \% ,$$

where n is the each point of the test in time t, N_*c*_ and N_*t*_ are the cell number in the control and in the culture incubated with toxicant, respectively, Cc and Ct are the chlorophyll a contents in the control and in the culture incubated with toxicant, respectively.

### Data Analysis

All data were expressed as mean ± standard error (S.E.M.). Analysis of variance (ANOVA) was used to compare significant differences in the growth inhibition rates of algae, and the combined effect of pH and phenanthrene was analyzed by Analysis of Covariance (ANCOVA). The phenanthrene dose causing 10% or 50% inhibition (EC_10_ or EC_50_) was calculated by linear regression of the inhibition percentage versus the logarithm of phenanthrene concentration^56^. Linear regression analysis was applied to evaluate the relationship between the cell count groups and the fluorescence measurement groups with GraphPad Prism 6.0. Values were considered significantly different when the probability (p) was less than 0.05.

## Data Availability

The datasets generated during and analyzed during the current study are available from the corresponding author on reasonable request.
